# Real-Time Pain Assessment from Electrodermal Activity Using Deep Learning

**DOI:** 10.3390/s26103020

**Published:** 2026-05-11

**Authors:** Calvin Joseph, Maryam Ghahramani, Raul Fernandez Rojas

**Affiliations:** Biosensing and Intelligent Systems (BioSIS) Lab, Centre for Intelligent Computing and Systems (CICS), University of Canberra, Canberra, ACT 2617, Australia

**Keywords:** pain assessment, continuous monitoring, electrodermal activity, real time monitoring, deep learning

## Abstract

Objective pain assessment remains a significant challenge in clinical and research settings due to the subjective nature of self-reported measures. Physiological signals, particularly electrodermal activity (EDA), have emerged as promising indicators of autonomic responses associated with pain. Although recent advances in deep learning have improved the modelling of complex biosignals, many existing approaches remain computationally demanding, limiting their applicability for real-time monitoring in wearable and embedded systems. This paper proposes a fully convolutional network (FCN) for automated pain recognition using EDA signals. The proposed model is designed to efficiently capture temporal patterns in physiological data while maintaining low computational complexity. The approach is evaluated on the AI4Pain dataset for three-class pain classification (No Pain, Low Pain, High Pain). Experimental results show that the proposed FCN achieves an accuracy of 79.23% in offline evaluation. Furthermore, the model enables real-time inference with a latency of 0.47 ms, achieving 73.14% accuracy during real-time operation. These results demonstrate that convolutional architectures can provide an effective balance between predictive performance and computational efficiency, supporting the development of real-time physiological pain monitoring systems using wearable sensing technologies.

## 1. Introduction

Pain is a complex, subjective experience that poses significant challenges in both clinical and research settings, particularly when it comes to objective assessment [1]. Traditional methods for evaluating pain often rely on self-reports, which, while valuable, are inherently limited by various external and internal factors [2]. Patient communication abilities, psychological state, cultural background, and the possibility of patients misrepresenting their pain levels can all influence these self-reported measures, introducing a level of variability and bias that complicates accurate pain assessment. This variability poses a considerable challenge for healthcare professionals, who must navigate these subjective reports to make critical decisions about pain management [3]. The impact of these limitations is significant, as inaccurate pain assessment often leads to suboptimal treatment strategies, prolonged suffering, and a decline in the overall quality of care. In clinical settings, where precise and timely interventions are crucial, there is a pressing need for objective pain assessment tools that can provide consistent and reliable measurements, thus enhancing the ability of healthcare professionals to manage pain effectively and improve patient outcomes.

Among various physiological signals, electrodermal activity (EDA) has emerged as a promising proxy for pain assessment [4]. EDA measures the electrical conductance of the skin, which varies with sweat gland activity and is influenced by emotional and physiological states [5]. This makes EDA especially valuable in capturing the body’s autonomic responses, providing a sensitive and reliable indicator of pain-related changes [6]. Compared to other biosignals such as photoplethysmography (PPG), electrocardiography (ECG), and electroencephalography (EEG), EDA presents several distinct advantages. It is less invasive, more cost-effective, less affected by motion artifacts, and easier to monitor continuously over time. Additionally, EDA is highly responsive to stress and arousal, making it particularly sensitive to pain-related changes in the autonomic nervous system [7]. Combined with advancements in wearable sensors and machine learning, the abilities of EDA to detect subtle physiological responses make it an excellent candidate for real-time, non-invasive pain monitoring. This approach offers more reliable and immediate insights into a patient’s pain experience compared to traditional methods. These qualities make EDA an important tool in the advancement of pain assessment technologies, particularly in contexts where continuous and unobtrusive monitoring is required. Furthermore, its ability to detect these subtle physiological responses, and with advancements in wearable sensors combined with machine learning, makes EDA an excellent candidate for real-time and non-invasive pain monitoring.

In recent years, advances in deep learning (DL) have enabled more accurate and efficient processing of complex physiological data. Traditional machine learning methods often struggle with the intricate and non-linear nature of physiological signals, requiring extensive feature engineering and domain expertise to achieve reliable results [8]. Unlike traditional approaches, DL models can automatically learn and extract relevant features from raw EDA data. This ability to capture intricate patterns and dependencies within the data has the potential for achieving more accurate and robust pain detection [9]. Furthermore, DL models, particularly those using architectures like Convolutional Neural Networks (CNNs), Recurrent Neural Networks (RNNs), and their variants, have demonstrated remarkable ability to automatically learn and extract features directly from raw or minimally-processed data [10]. This has led to substantial improvements in tasks such as classification, prediction, and anomaly detection across various biosignal analysis tasks, including ECG for arrhythmia detection [11], EEG for seizure prediction [12], and EMG for gesture recognition [13]. The ability of DL models to handle the temporal and spatial complexities inherent in EDA signals, with minimal preprocessing, not only streamlines the analytical process but also opens new avenues for real-time monitoring, making it a potential tool in pain assessment tasks.

While advanced DL models can significantly improve pain detection accuracy, their practical application in real-time clinical settings requires systems that can effectively support clinical decision-making. Such systems translate complex computational outputs into actionable insights for healthcare professionals [14]. These AI-based tools can facilitate real-time monitoring, allowing clinicians to quickly assess pain levels and make data-driven decisions with confidence [15]. Moreover, hospitals routinely collect vast amounts of physiological data, including sensor readings and vital signs like heart rate, skin conductance, and oxygen levels [16]. However, these data are often underutilised in clinical decision-making, particularly when it comes to AI-based pain assessment. Leveraging this existing data and integrating it into intelligent, DL-based systems can significantly enhance pain management by offering clinicians more objective and timely insights. The development of these AI-based platforms that utilise routinely collected physiological data is crucial to bridging the gap between cutting-edge artificial intelligence and its real-world application in healthcare settings.

Despite these advances, many DL approaches for physiological pain recognition remain computationally demanding. This limitation restricts their suitability for real-time monitoring in wearable and embedded systems. To address this challenge, this paper proposes an efficient FCN for real-time pain recognition using EDA signals. The proposed approach is evaluated on the AI4Pain dataset for three-class pain classification, demonstrating that efficient convolutional architectures can achieve competitive predictive performance while enabling low-latency inference suitable for real-time applications. The main contributions of this work are as follows:

1. A task-specific adaptation and optimisation of a fully convolutional network for temporal modelling of EDA signals, enabling effective extraction of autonomic dynamics for pain assessment.
2. A real-time physiological pain estimation framework that integrates the proposed model within a sliding-window inference pipeline, achieving low-latency prediction (0.47 ms) and continuous pain estimation on unseen subjects.
3. A comprehensive evaluation on the AI4Pain dataset for three-class pain classification (No Pain, Low Pain, High Pain), demonstrating that the proposed FCN achieves competitive performance (79.23% accuracy) using a single physiological modality (EDA), while maintaining a favourable trade-off between accuracy and computational efficiency.

It is important to note that this work does not propose a novel FCN architecture; rather, it focuses on the adaptation, optimisation, and real-time deployment of FCNs for physiological time-series modelling in pain assessment. These results highlight the potential of DL architectures to enable practical real-time pain monitoring using wearable physiological sensing technologies.

## 2. Methods

### 2.1. Experimental and Dataset

The dataset utilised in this study is based on the AI4Pain Grand Challenge dataset [17], with the methodology and experimental procedure previously reported in [18]. For this study, only the EDA data are used for pain assessment.

Sixty-five (*n* = 65) participants, including 23 females, took part in the experiment. Their ages ranged from 17 to 52 years (mean 29.06 yr, standard deviation 8.28 yr). No participants reported a prior history of neurological or psychiatric disorder, a current unstable medical condition, chronic pain, regularly taking medications or being under medication at the time of testing. Participants were given a detailed explanation of the experimental procedures upon their arrival. Written informed consent was obtained before the start of the experiment. The experimental procedures involving human subjects described in this paper were approved by the University of Canberra’s Human Ethics Committee (number: 11837).

EDA data were recorded using a Biosignal Plux (Lisbon, Portugal) sensor, sampled at 100 Hz. EDA signals were acquired using gelled self-adhesive disposable Ag/AgCl electrodes, which are commonly used for skin conductance measurements due to their stable electrode–skin interface and low impedance characteristics. The electrodes were placed on the proximal phalanges of the index and middle fingers, where sweat gland density is high, ensuring reliable capture of autonomic responses. EDA signals reflect sympathetic nervous system activity through changes in skin conductance, primarily associated with sweat gland activation. These signals are typically low-frequency in nature (below 3 Hz), in contrast to other physiological signals such as surface electromyography (sEMG), which capture muscle activation and operate over higher frequency ranges (approximately 20–450 Hz). Consequently, EDA acquisition relies on conductance-based electrodes and requires different signal processing approaches compared to bioelectric signal measurements such as EMG.

For pain induction, transcutaneous electrical nerve stimulation (TENS) electrodes (Medihightec Medical Co., Ltd., New Taipei City, Taiwan) were placed on the inner forearm and on the back of the hand. While EDA signals were recorded from a fixed location on the hand, pain stimuli were applied at different anatomical sites using the TENS device. This design enables future investigation into whether physiological responses can be leveraged, in combination with additional sensing modalities, to infer the location of pain, although the present study focuses solely on pain intensity classification. To prevent habituation and reduce confounding effects due to order effects, the location and intensity of the pain stimulus were counterbalanced across trials [19]. The stimulation parameters were calibrated individually to account for differences in pain sensitivity, ensuring reproducible and controlled experimental conditions. As a commercial system with predefined stimulation modes, the exact waveform parameters are device-controlled; therefore, the stimulation intensity (ranging from 0 to 100 mA) was individually adjusted for each participant to elicit distinct low and high pain levels while remaining within safe and tolerable limits. Each stimulation trial lasted approximately 10 s, followed by a rest period to allow physiological recovery. This protocol ensured consistent and repeatable induction of pain levels across participants.

**Figure 1:**
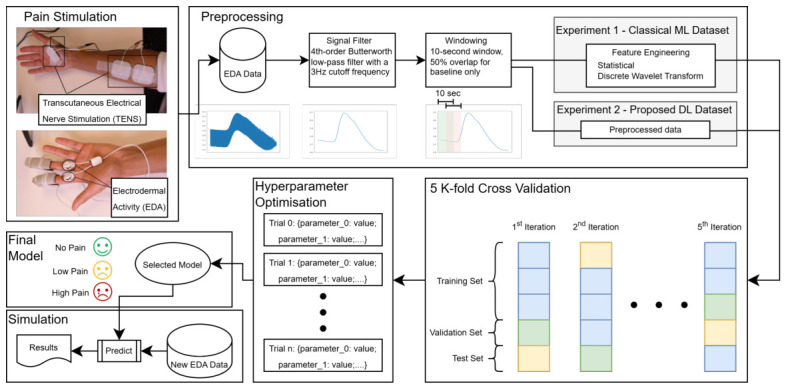
Overview of the propose methodology for automatic pain estimation using electrodermal activity signals. The framework includes data acquisition, preprocessing, machine learning and deep learning model development, cross-validation, hyperparameter optimisation, and deployment within a GUI-based real-time pain estimation system.

The AI4Pain dataset is stratified into three distinct categories of varying levels of pain intensity: *No Pain*, *Low Pain*, and *High Pain*. Specifically, the dataset consists of 65 instances (each lasting 60 s) of *No Pain*, 780 instances of *Low Pain* (each lasting 10 s), and 780 instances of *High Pain* (each lasting 10 s). The *No Pain* category includes instances from the baseline period at the start of each experiment, providing physiological data for comparison with pain-induced responses. The *Low Pain* category comprises instances of mild pain based on the pain threshold test, capturing subtle neurological and behavioural changes in the data. Finally, the *High Pain* category consists of instances where subjects experienced significant pain, based on the pain tolerance test, leading to notable physiological and behavioural responses in the EDA data. Figure 1 presents an overview of the proposed methodology, including data acquisition, preprocessing, cross validation, hyperparameter optimisation, model development, and real-time pain estimation. In addition, Table 1 summarises the electrodermal activity (EDA) data obtained from the AI4Pain dataset.

### 2.2. Preprocessing

To ensure balanced representation across pain conditions, a targeted balancing strategy was applied exclusively to the baseline (i.e., *No Pain*) recordings. Each 60 s baseline segment was processed using a 50% overlapping windowing scheme, producing eleven 10 s windows per participant and yielding a total of 715 baseline samples (11 × 65 participants). This approach increased the number of baseline instances while preserving temporal continuity and variability within non-painful states. In contrast, the *Low Pain* and *High Pain* recordings were maintained in their original form as non-overlapping 10s windows, resulting in 780 samples (12 × 65 participants) for each pain class. The selective overlapping method applied to the *No Pain* functioned as a class-balancing procedure rather than a general data-augmentation technique, ensuring comparable sample sizes across conditions without artificially inflating the pain data.

In addition, the raw EDA signals were preprocessed to minimise high-frequency noise and motion artefacts prior to model training. A 4th-order low-pass Butterworth filter with a 3 Hz cut-off frequency was applied [20]. The EDA signals were not decomposed into phasic and tonic components to preserve the full temporal and spectral content of the physiological responses. This approach retains both short-term sympathetic activations and longer-term arousal trends, enabling the learning model to capture a broader representation of pain-related autonomic dynamics. Additionally, avoiding explicit decomposition prevents potential distortions introduced by deconvolution-based separation methods, thereby maintaining the physiological integrity of the recorded EDA signal [5,21].

### 2.3. Learning Models

This study presents the application of DL for the design of a real-time pain assessment system. To this end, four of the most successful DL models for time series classification were investigated [22,23,24], these are: CNN, long short-term memory (LSTM), CNN-LSTM, fully convolutional networks (FCN), and transformers. Two experiments were conducted to evaluate and compare model performance. The first experiment established baseline reference values using classical machine learning algorithms trained on handcrafted features. The second experiment evaluated the DL models directly on the EDA data, without feature engineering, to assess their end-to-end learning capability. It was hypothesised that DL models would outperform classical machine learning approaches, as they can learn hierarchical, non-linear representations and capture subtle temporal patterns associated with pain responses directly from EDA signals. Finally, the best-performing model was integrated into a graphical user interface (GUI) designed for real-time pain estimation, which was subsequently validated on an independent dataset comprising 15 unseen subjects.

#### 2.3.1. Reference Models

To establish baseline performance and characterise the advantages of the proposed DL models, several classical machine learning classifiers were implemented. These included both linear and non-linear approaches: linear discriminant analysis (LDA), logistic regression (LR), support vector machines (SVM), AdaBoost, and gradient boosting (GBoost). Each classifier was optimised through Bayesian optimisation to identify the best-performing hyperparameters [25]. These parameters are the optimisation algorithms (i.e., solver) [singular value decomposition, least squares solution, eigenvalue decomposition] for LDA. The regularisation methods [l1, l2, elasticnet, no regularisation] and their strength C [floats from 0.01 to 100.0] of LR, and adding different solvers [L-BFGS-B, Newton conjugate gradient, Cholesky decomposition, stochastic average gradient, stochastic average gradient accelerated] [26,27,28]. For SVM, we optimised the kernel type [linear, polynomial, and radial basis function (RBF)]. If RBF was selected for a given trial, then the degree [integers of 1 to 5] and the gamma coefficient [0.0001, 0.001, 0.01, 0.1] were optimised. Different learning rates [floats of 0.0001 to 0.9] and numbers of estimators [integers of 10 to 500] for each boosting algorithm were searched, and the criterion was [Friedman mean-squared-error, squared-error] for GBoost.

The hand-crafted feature set was derived from widely used statistical and signal processing measures. From each 10-s window, six time-domain features were extracted: mean, standard deviation, skew, kurtosis, minimum, and maximum. In addition, the EDA signals were decomposed using the discrete wavelet transform (DWT) with Daubechies (db4) wavelets, capturing approximate and detailed coefficients up to five levels of decomposition, which is sufficient to capture both tonic and phasic variations in the 0–3 Hz range. For each level, the same six statistical features were computed, resulting in a total of 36 features per window when combined with the time-domain metrics. Finally, principal component analysis (PCA) was applied to the combined feature space to reduce dimensionality and retain, based on the scree plot, the 12 most informative components for model training. These extracted features were subsequently used as input to the baseline machine-learning classifiers described above.

#### 2.3.2. Proposed Models

To evaluate the performance of DL in continuous pain recognition from EDA signals, five representative architectures were implemented. These architectures were selected to represent the main paradigms of DL for time-series analysis, spatial feature extraction (CNN, FCN), temporal sequence modelling (LSTM), hybrid feature–temporal fusion (CNN-LSTM), and self-attention mechanisms (Transformer). To complement the comparison across modelling paradigms, a MobileNetV3 architecture [29] was additionally implemented as a representative lightweight model. This inclusion allows evaluation of efficiency-oriented design strategies based on depthwise separable convolutions, without aiming for exhaustive benchmarking of all lightweight variants. Each model was trained and evaluated under identical conditions using the same preprocessed and windowed EDA data to ensure fair comparison. This methodological consistency allowed performance differences to be attributed to the architectural capabilities of each model rather than training or preprocessing bias.

Similar to the reference models, hyperparameter optimisation was performed via Bayesian optimisation for all models [25]. For all DL architectures, the optimisation space included the choice of optimiser algorithm [Adam, AdamW, root-mean-square propagation (RMSprop), support gradient descent (SGD)], learning rate (floats of 1 × 10^−5^ to 1 × 10^−1^), and learning rate scheduler [no scheduler, linear decrease (optimised the step size between 2 and 200), exponential decrease, reduce on plateau].

The architecture of the CNN implemented in this study has the following components. All of the convolutional-based networks have an input shape of [B,C,S], where *B* denotes the batch size, *C* the number of channels, and *S* the temporal length of the signal. In our case, C=1 because a single EDA signal is used, and S=1000 samples, corresponding to 10 s segments recorded at 100 Hz. The CNN is composed of 1D convolutional layers search space, integers 1 to 3, with each layer having output features integers 4 to 128 and kernel size integers 2 to 4, followed by an activation function none, ReLU, sigmoid, tanh. A pooling layer none, max, average is applied after each convolutional block, with kernel size integers of 2 to 6. Batch normalisation is incorporated to stabilise training and improve convergence. The convolutional backbone concludes with a flattening operation. Following the convolutional feature extractor, a fully connected classifier is appended, with output size integers of 3 to 128. In all models, the final output layer has an output size equal to the class number (three in this study).

A recurrent architecture was implemented based on one or more stack integers of 1 to 5 LSTM layers. When multiple LSTM layers are stacked, dropout regularisation floats of 0.0 to 0.75 are applied between successive layers to mitigate overfitting. Additional LSTM hyperparameters are also optimised, including the hidden state dimensionality integers of 1 to 1000 and the processing directionality unidirectional, bidirectional. The recurrent backbone is followed by a fully connected classification head and the final output layer.

The CNN–LSTM architecture builds upon the previously described convolutional and recurrent modules, adopting the same design principles and hyperparameter search space. In this hybrid configuration, the 1D CNN is first used as a feature extractor to learn local temporal patterns from the input signal, and its output is subsequently processed by the LSTM layers to model longer-term temporal dependencies. A transition layer is introduced between the convolutional and recurrent components to adapt the CNN feature maps to the sequential input format required by the LSTM. Depending on the configuration, this transition is implemented either through a flattening operation or through a temporal pooling layer max or average pooling applied along the last dimension of the CNN output to reduce sequence length and computational complexity. The resulting feature sequence is then fed into one or more LSTM layers, followed by a fully connected classification head that produces the final class logits.

The FCN architecture, originally introduced for dense prediction tasks in computer vision [30], is conceptually similar to the previously described CNN models and follows the same hyperparameter search space for the convolutional backbone. Specifically, the network consists of 1D convolutional layer integers 1 to 3, each producing output feature map integers 4 to 128 with kernel size integers 2 to 4. Each convolutional layer is followed by batch normalisation and an activation function, in which the search spaces are similar to our CNN implementation, to improve training stability and introduce non-linearity. After the convolutional blocks, a global or local average pooling layer is applied, with the pooling kernel size treated as a hyperparameter with integers 2 to 16, in order to aggregate temporal features and reduce the dimensionality of the representation. The resulting feature vector is then flattened and passed to a fully connected classification layer that maps the learned representation to the final output space, producing the class logits.

A lightweight encoder-only Transformer architecture was implemented. It was followed by a fully connected classification head that maps the learned representations to the output space. The model consists of a stack of Transformer encoder blocks, where the number of encoder layers is treated as a hyperparameter with integers 1 to 12. Dropout regularisation with floats 0.1 to 0.3 is applied between encoder layers to improve generalisation. Within each encoder block, the multi-head self-attention module is parameterised by the number of attention heads with integers 4 to 12. The position-wise feedforward network dimensionality is also treated as a hyperparameter and is defined as a multiple of the model embedding dimension, i.e.,

$$d_{\mathrm{ff}}=d_{\mathrm{model}}\times n_{\mathrm{ff}},$$

where $d_{\mathrm{model}}$ corresponds to the input feature dimension and $n_{\mathrm{ff}}$ is the searched integers, 2 to 4. This configuration allows the model capacity to scale proportionally with the input representation while controlling computational complexity. The final representation produced by the encoder stack is passed to a fully connected layer that outputs the class logits for classification.

#### 2.3.3. Model Evaluation

For model evaluation, a strict subject-wise five-fold cross-validation (5F CV) protocol was employed to prevent subject leakage between training and testing sets. All samples from each subject were assigned exclusively to a single fold, ensuring that no subject contributed data to both training and testing sets within any cross-validation iteration. The dataset was divided into five folds, each containing data from thirteen distinct subjects, with subject identities preserved across folds. All models were trained and evaluated using the same fold partitions, which were randomly generated at the start of the experiment. For each cross-validation iteration, three folds were used for training, one fold for validation, and one fold for testing. Leave-one-subject-out CV was not used in this study due to its substantially higher computational cost and training time, which would have been prohibitive given the large number of DL trials and hyperparameter optimisation runs. Model training and optimisation were performed on an Intel Xeon Silver 4116 CPU and NVIDIA A100 GPU to enable efficient multiprocessing and accelerated computation.

Hyperparameter optimisation was conducted using Optuna [25], within the 5F CV framework. For each fold, Optuna automatically searched for the optimal configuration yielding the highest validation accuracy. Each Optuna trial instantiated a model with a unique set of hyperparameters, and up to 1000 trials were executed per fold, resulting in 1000 candidate models. During training, each model was trained for a maximum of 3000 epochs, with early stopping applied if validation accuracy failed to improve for 500 consecutive epochs. The Adam optimiser and cross-entropy loss function were used for weight updates. For each trial, the checkpoint corresponding to the highest validation accuracy was retained. The configuration yielding the best validation performance across all trials was then selected and evaluated once on the held-out test set to obtain the final performance estimates.

#### 2.3.4. Real-Time Pain Estimation

Finally, the best-performing DL model was integrated into a custom GUI designed for real-time pain estimation. To ensure experimental consistency, this validation phase followed exactly the same recording protocol and sensor configuration used in the AI4Pain dataset, employing BioSignalPlux sensors for physiological data acquisition. The GUI was evaluated on an independent dataset comprising 15 unseen subjects (7 females), who underwent the same pain calibration, induction, and baseline procedures. The average age on this sample is 27.06 year old with a standard deviation of 3.96. This external validation step assessed the model’s real-time inference capability and generalisability across participants, demonstrating its practical applicability for continuous pain monitoring in controlled laboratory environments.

The real-time estimation procedure is summarised in Algorithm 1. The algorithm continuously acquires EDA samples, constructs a sliding window of fixed length (1000 samples), and performs inference using a trained classifier. A new prediction is generated every 100 samples after the initial window is filled. In addition, the algorithm is independent of the specific classifier and can operate with any trained model. The final model used in the GUI implementation is selected based on the performance comparison presented in Section 3.
**Algorithm 1** Real-time pain estimation algorithm.samples ←∅window_ready ← Falsecounter ←0**while** streaming **do**    x← acquire sample from EDA sensor    append *x* to samples    **if** ¬window_ready **then**      **if** |samples|=1000 **then**         window_ready ← True         w←ButterworthFilter(samples)         p←Model(w)         predicted_pain ←argmax(p)         confidence ←max(p)         display(predicted_pain, confidence)         counter ←0      **end if**    **else**      remove oldest sample from samples      counter ←counter+1      **if** counter=100 **then**         w←ButterworthFilter(samples)         p←Model(w)         predicted_pain ←argmax(p)         confidence ←max(p)         display(predicted_pain, confidence)         counter ←0      **end if**    **end if****end while**

## 3. Results

In this section, the results are presented in two stages. First, the performance of the proposed DL (CNN, LSTM, CNN-LSTM, FCN, Tranformer) models is compared against conventional machine learning (e.g., LDA, LR, SVM, ADABOOST, GBOOST) and feature-based methods. Second, the best-performing model is deployed within a GUI and evaluated on an independent dataset of 15 previously unseen participants collected using the same experimental protocol as the AI4Pain dataset.

### 3.1. Performance of the Machine Learning Models

To evaluate the performance of the DL models, several widely used ML models were investigated. Table 2 summarises the optimal hyperparameters identified for each model. Hyperparameter optimisation was performed using Bayesian optimisation to identify the configurations that maximise classification performance.

**Figure 2:**
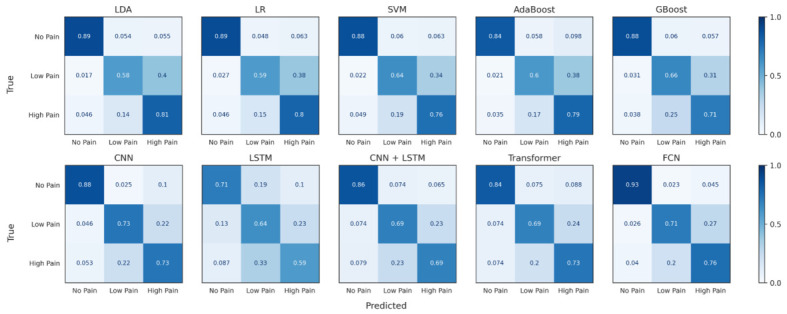
Normalised confusion matrices for all evaluated classical machine learning and DL models, showing the class-wise prediction performance for the three pain levels (No Pain, Low Pain, High Pain).

Table 3 summarises the performance of the evaluated machine learning models based on the median-performing fold of the cross-validation experiments. Overall, the linear models, including Linear Discriminant Analysis (LDA), Logistic Regression (LR), and Support Vector Machines (SVM), achieved comparable performance, each reaching an accuracy and F1-score of approximately 0.75 with low variability across folds. Among these models, LDA and LR achieved the highest weighted precision (0.77 ± 0.02), while SVM obtained slightly lower precision (0.76 ± 0.03). The ensemble-based approaches, AdaBoost and Gradient Boosting (GBoost), produced similar but marginally lower classification performance, with accuracies of 0.74. In terms of computational efficiency, the linear models exhibited extremely fast inference times (≤0.01 ms), while GBoost required slightly longer inference (0.06 ms), and AdaBoost showed the highest inference time among the evaluated ML methods (0.73 ms). In addition, the confusion matrices for the evaluated classical ML models are shown in Figure 2. These results indicate that traditional machine learning models provide consistent classification performance with minimal computational overhead, establishing a strong baseline for comparison with the DL approaches.

### 3.2. Performance of the DL Models

The final optimised hyperparameters for the evaluated DL models, including CNN, LSTM, CNN–LSTM, Transformer, and FCN architectures, are summarised in Table 4. The selected configurations correspond to the best-performing settings obtained from the defined hyperparameter search space during the optimisation process.

In addition, Table 5 presents the performance of the evaluated DL architectures. To provide a representative comparison across models while avoiding optimistic or pessimistic cases, the results reported in Table 5 correspond to the median-performing fold from the cross-validation experiments. The performance metrics are reported as the mean and standard deviation across all folds. The FCN achieved the best overall performance, obtaining an accuracy and F1-score of 0.79 (±0.03) and a weighted precision of 0.80 (±0.03). The CNN model also demonstrated strong performance with an accuracy of 0.77 (±0.04), indicating that convolutional architectures are effective for capturing discriminative temporal patterns in the physiological signals. In contrast, the standalone LSTM model achieved the lowest performance (0.64 ± 0.11), suggesting that recurrent architectures alone may be less effective at extracting robust features from the input sequences. The hybrid CNN–LSTM and Transformer models achieved comparable performance (0.74 accuracy), although they introduced higher computational complexity. In addition, a MobileNetV3 architecture adapted to 1D signals was evaluated as a representative lightweight model, achieving an accuracy of 0.75 (±0.01), F1-score of 0.75 (±0.01), and a weighted precision of 0.75 (±0.01), which is lower than the performance obtained with the proposed FCN. Finally, the confusion matrices for the evaluated DL models are shown in Figure 2, illustrating the class-wise prediction behaviour for the three pain levels; for clarity, confusion matrices are reported only for the primary models, while the MobileNetV3 results are summarised using overall performance metrics above.

### 3.3. Computational Efficiency

In addition to predictive performance, computational efficiency is an important consideration for real-time deployment. Therefore, the inference cost and computational complexity of both machine learning and DL models were analysed. Models with high computational complexity may achieve strong classification accuracy but can be unsuitable for continuous real-time inference due to increased processing requirements and latency. Therefore, the computational characteristics of the evaluated machine learning and DL models were analysed in terms of inference time and computational complexity. This analysis provides insight into the trade-offs between model performance and computational cost, which is particularly important for systems designed to operate in real-time environments. Based on these considerations, the most suitable architecture was selected for implementation in the GUI to enable continuous real-time estimation.

The computational cost of the evaluated classical ML models is summarised in Table 6. The inference time of all classifiers was very low, with Logistic Regression (0.00 ms), LDA (0.01 ms), and SVM (0.01 ms) being the fastest, while AdaBoost showed the highest inference cost (0.73 ms). The dominant computational component corresponded to the feature extraction stage, which required 3.77 ms per window on average across all windows from the 65 subjects. The total processing time was computed as the sum of the feature extraction cost and the model inference time. Under this criterion, Logistic Regression achieved the lowest total computational time (3.77 ms), followed closely by LDA and SVM (3.78 ms), whereas AdaBoost showed the highest total cost (4.50 ms). Overall, the total latency of all models remained in the millisecond range, with the feature extraction stage accounting for the largest portion of the computational cost.

The computational efficiency of the evaluated DL architectures, summarised in Table 7, reveals substantial differences in both computational cost and inference latency. Unlike the classical machine learning pipeline, where feature extraction is performed separately, feature learning in DL models is integrated within the network and therefore included in the reported inference latency. The CNN model required the lowest computational cost (6.43 MFLOPs), while the FCN maintained moderate complexity (52.25 MFLOPs) with a fast inference time of 0.47 ms. In contrast, the CNN–LSTM architecture incurred substantially higher computational cost (3659.15 MFLOPs), and the Transformer exhibited the longest inference time (2.29 ms). To further evaluate lightweight architectures, a MobileNetV3 model adapted to 1D signals was implemented, with a computational cost of 165.76 MFLOPs and inference time of 7.98 ms. Compared to the proposed FCN (0.79 accuracy; 52.25 MFLOPs), this indicates a less favourable efficiency–performance trade-off. Overall, these results indicate that convolution-based architectures, particularly FCN, provide a favourable balance between classification performance and computational efficiency for this task. Based on this trade-off, the FCN model was selected for implementation in the GUI to enable continuous real-time estimation.

It is important to distinguish between model inference time and total processing latency, while classical machine learning models exhibit extremely low inference times, they require a separate feature extraction stage, which introduces additional computational cost. In this study, feature extraction required approximately 3.77 ms per window, dominating the overall processing time. In contrast, deep learning models perform feature extraction and classification jointly within the network, resulting in lower end-to-end latency. Therefore, although the FCN has a higher standalone inference time (0.47 ms), it achieves significantly lower total processing time compared to traditional pipelines, while also providing improved classification performance.

The final FCN architecture used in the real-time GUI is summarised in Table 8. The network receives a single-channel temporal window of 1000 samples corresponding to the preprocessed physiological signal. The first convolutional block (CL0) applies one-dimensional convolution, batch normalisation, and ReLU activation to produce 71 feature maps. A second convolutional block (CL1) further refines the representation using convolution, batch normalisation, and Tanh activation, increasing the number of feature maps to 120. The feature maps are then downsampled using average pooling, and the resulting representation is flattened into a feature vector of size 39,840 for the final classification layer. This architecture was selected for the GUI-based real-time analysis due to its favourable balance between classification performance and computational efficiency among the evaluated models.

### 3.4. GUI Real-Time Estimation

**Figure 3:**
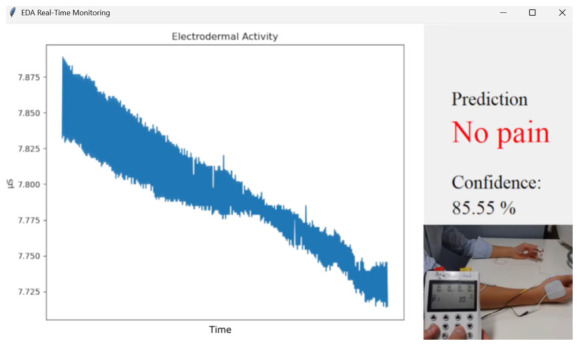
Example of the GUI during a TENS stimulation experiment. The left panel shows the EDA signal, while the right panel displays the predicted pain class together with the confidence level expressed as a percentage.

The FCN model was integrated into a GUI to enable continuous pain level estimation. To provide additional interpretability during real-time operation, a confidence level was computed from the output probabilities of the final softmax layer. The confidence value was defined as the maximum probability among the three output neurons, corresponding to the predicted pain class. The confidence level provides a measure of prediction certainty, enabling the system to quantify the reliability of the estimated pain class during real-time inference. An example of the GUI running during an experiment is shown in Figure 3.

**Figure 4:**
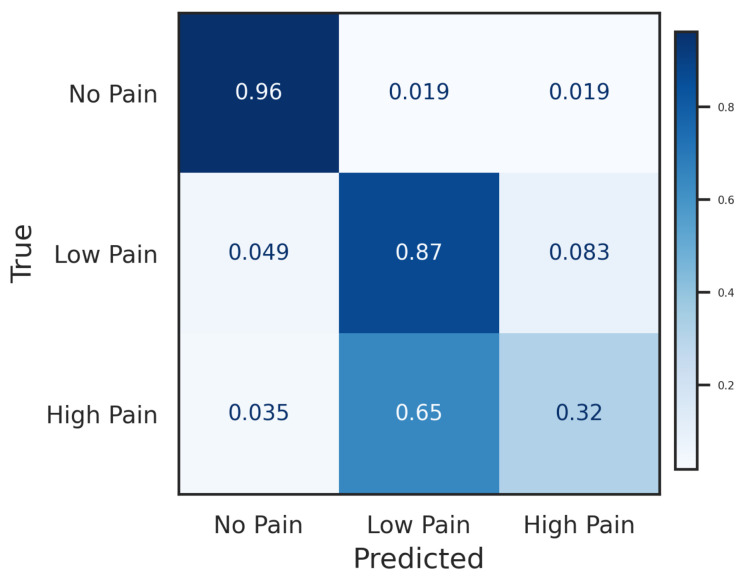
Confusion matrix obtained during the GUI simulation phase. Rows represent the true labels and columns represent the predicted labels for the three pain categories (No Pain, Low Pain, High Pain). Values are normalised per class.

**Figure 5:**
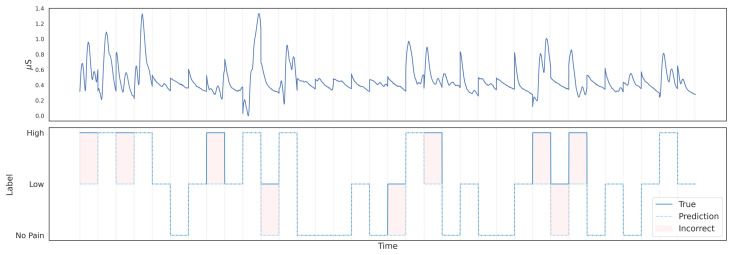
Example of temporal pain estimation obtained from the GUI during a TENS stimulation experiment. The top plot represents a calibrated EDA signal during the stimulus period per window, while the bottom plot shows the ground-truth against model prediction. The solid line represents the ground-truth labels, while the dashed line shows the model predictions over time. The three label levels correspond to No Pain, Low, and High. For visual clarity, the plot does not display every individual prediction generated by the system.

The proposed FCN model was evaluated in the GUI-based real-time estimation framework to assess its performance during continuous operation. The model achieved an accuracy of 73.14%, with weighted precision of 75.8%, and F1-score of 70.43%. The confusion matrix obtained during the GUI simulation phase is shown in Figure 4, where the model correctly classified most of the *No Pain* and *Low Pain* samples, while a larger number of misclassifications occurred for the *High Pain* category, which was frequently predicted as *Low Pain*. This behaviour suggests that the model can reliably distinguish the absence of pain and moderate stimulation levels, but transitions between moderate and high pain remain more challenging during continuous estimation. In addition, Figure 5 shows an example of the temporal evolution of the predicted pain labels compared with the ground-truth annotations during the TENS stimulation experiment, illustrating the ability of the model to track changes in pain level over time.

## 4. Discussion

The results confirm that physiological responses contain sufficient information to distinguish between different pain levels using both ML and DL models. Pain stimuli produce measurable changes in sympathetic activity, including variations in skin conductance, heart rate, and respiration, which have long been used as objective indicators of nociceptive processing [31]. These physiological changes evolve over time and therefore require models capable of capturing temporal dynamics, which explains why both feature-based and DL approaches achieved meaningful performance [32]. The consistent performance across different classifiers suggests that the discriminative information is embedded in the temporal dynamics of the physiological signals rather than in a specific modelling approach. This behaviour is particularly relevant for EDA, which directly reflects sympathetic activation and is known to increase with stimulus intensity, although the response is gradual and subject-dependent rather than strictly discrete. From a physiological perspective, the placement of EDA electrodes on the proximal phalanges of the index and middle fingers is motivated by the high density of eccrine sweat glands in the palmar skin, which are strongly innervated by the sympathetic nervous system [33]. This configuration enables high-sensitivity electrodermal responses to pain-related autonomic activation and supports reliable recording of EDA changes during the stimulation protocol. Because EDA signals contain tonic and phasic components with non-linear temporal variations, traditional feature-based methods can capture global statistical properties, while DL models are better suited to modelling complex temporal patterns [9]. These results indicate that improvements in automatic pain estimation using EDA may depend not only on the classifier architecture but also on how temporal information is represented and extracted from the physiological signal.

Class-wise performance indicates that pain levels can be separated reliably offline but become less distinct during real-time estimation. The confusion matrices in Figure 2 show that most models achieve clear separation between the three pain classes, with relatively few misclassifications in the offline evaluation. However, additional errors appear in the GUI real-time results, where predictions are computed continuously from sliding windows of EDA data. This behaviour is consistent with the physiological properties of EDA, which reflects sympathetic nervous system activation and increases progressively with stimulus intensity rather than producing discrete response levels [31]. Experimental studies have shown that skin conductance responses grow with painful stimulation but often exhibit overlapping ranges across different intensities, especially when stimuli are close in magnitude, making multi-class discrimination inherently difficult [34]. Continuous estimation inherently captures transitions between stimulation levels, increasing the likelihood of intermediate predictions despite strong offline performance. Unlike offline evaluation on stable segments, real-time sliding windows include transitional states, leading to overlapping physiological responses between Low Pain and High Pain and, consequently, increased classification ambiguity. This effect is further amplified by inter-subject variability, habituation, and temporal smoothing introduced by the sliding-window mechanism. This limitation can be mitigated by refining the preprocessing stage for real-time data to better isolate stable signal segments and reduce temporal interference and artefacts that are less prominent under controlled experimental conditions. In addition, EDA responses are strongly influenced by individual autonomic reactivity, habituation, and sensitisation effects [35], which can cause different signal amplitudes for the same stimulus and reduce class separability when using a single global model. The pattern observed in the confusion matrices therefore reflects a known limitation of EDA-based pain estimation rather than a weakness of a particular classifier. This highlights the importance of evaluating pain recognition models under real-time conditions [36], as offline accuracy alone may overestimate the performance achievable in practical monitoring systems.

The FCN provided the best trade-off between classification performance and inference time, which enabled its integration into the GUI for continuous real-time estimation. Real-time physiological monitoring requires models capable of processing streaming data with low latency, particularly when predictions must be updated continuously using sliding windows of biosignals. Previous work in physiological computing has emphasised that online systems must balance predictive accuracy and computational load to ensure stable operation during continuous acquisition [36,37]. In the context of time-series analysis, convolution-based architectures have been shown to achieve efficient inference while preserving the ability to capture relevant temporal patterns, making them well suited for real-time applications [38]. Unlike recurrent or hybrid architectures, FCNs rely exclusively on convolutional operations combined with global average pooling, enabling hierarchical feature extraction with a relatively small number of parameters [39]. This structure allows FCNs to model discriminative local temporal patterns in physiological signals without the computational overhead associated with recurrent processing, resulting in fast inference and moderate complexity while maintaining strong classification performance [40,41]. The results obtained in the GUI experiments demonstrate that, for real-time physiological monitoring, selecting an appropriate architecture requires considering both predictive accuracy and computational efficiency, as models with higher theoretical capacity do not necessarily provide practical advantages when deployed in continuous estimation frameworks.

Recent studies using the AI4Pain dataset and related physiological pain-estimation frameworks report comparable performance levels. The AI4Pain dataset was specifically designed to support research on automatic pain recognition using physiological recordings (EDA, BVP, RESP, SpO_2_) collected during controlled stimulation experiments, and has been used in several recent studies to evaluate machine-learning and deep-learning approaches for three-level pain classification. The comparison in Table 9 shows that among the methods evaluated on the hidden test set, the best-performing approach achieved an accuracy of 75.83%, demonstrating the potential of transformer-based multimodal fusion models. However, several studies report results obtained using cross-validation protocols (e.g., LOSO or k-fold CV), which are not directly comparable with hidden test set evaluations; the table is therefore intended to provide an overview of the methods, sensor modalities, and performance ranges reported in the literature. Despite these differences in evaluation protocols, the comparison highlights the increasing interest in DL architectures for physiological pain assessment. In this context, the proposed FCN achieves an accuracy of 79.23%, outperforming previously reported results obtained using cross-validation while relying on a single physiological modality (EDA). This result suggests that efficient temporal feature learning with convolutional architectures can provide strong performance for pain recognition, even without relying on complex multimodal fusion schemes.

Real-time physiological pain assessment remains an emerging research area, with relatively few studies explicitly addressing both predictive performance and computational efficiency. As shown in Table 10, most existing approaches rely on EDA as the primary sensing modality, reflecting its strong association with autonomic responses to nociceptive stimuli. Several studies focus on binary pain detection (No Pain vs Pain), while fewer works investigate multiclass pain recognition. In terms of modelling strategies, prior work includes recurrent architectures such as stacked LSTMs, CNNs, attention-based models, and transformer-based approaches. However, many studies do not report inference latency, making it difficult to assess their suitability for real-time deployment. Among the works that provide computational efficiency metrics, the MDNet model reports inference times on the order of hundreds of milliseconds and was demonstrated on an embedded platform using a Raspberry Pi 5, highlighting its applicability for edge-based pain monitoring systems. Transformer-based approaches, in contrast, may require several seconds per prediction. In comparison, the proposed FCN achieves a latency of 0.47 ms while maintaining competitive classification performance (73.14% accuracy) for three-class pain recognition. These results indicate that low-latency convolutional architectures can offer an effective balance between predictive performance and computational efficiency, making them particularly suitable for real-time physiological pain monitoring in embedded or wearable systems.

Several experimental constraints should be considered when interpreting the present results. First, the experiments were conducted under controlled laboratory conditions using induced pain, which may not fully reflect the complexity of clinical or chronic pain. Previous studies have shown that physiological responses to experimentally evoked stimuli can differ from responses observed in real clinical settings, where pain is influenced by cognitive, emotional, and contextual factors [1]. Second, the proposed framework relies on a single physiological modality, namely, EDA, which limits the amount of information available for classification. Recent reviews on automatic pain assessment indicate that multimodal approaches combining several biosignals often provide more robust and reliable performance than unimodal systems, particularly in multi-class problems [60,61]. Another limitation is the use of a global model trained across all subjects, which may reduce accuracy due to large inter-individual differences in autonomic responses. Variability in EDA related to age, stress, and baseline sympathetic tone has been reported as a major challenge for physiological monitoring systems [4]. In addition, a limitation of this study is that the proposed framework was evaluated on a single dataset (AI4Pain). Although this dataset provides controlled experimental conditions and supports three-class pain classification with consistent temporal windows, future work should investigate cross-dataset generalisation. Finally, the use of fixed-length sliding windows introduces temporal smoothing, which can reduce sensitivity to rapid changes in stimulation during real-time estimation. These factors suggest that the current results should be interpreted as a proof of concept rather than a complete solution for practical pain monitoring.

Future work should prioritise multimodal, adaptive, and clinically grounded pain estimation. Although the present results show that EDA alone can support automatic pain estimation, recent work indicates that multimodal physiological modelling can improve robustness by combining complementary information across autonomic signals. For example, Thiam et al. [62] used multimodal physiological signals for pain intensity assessment and showed the value of combining signals within deep-learning frameworks, while recent clinical-facing work has emphasised integrating biosignal-based automated pain assessment with richer patient information and real-world workflows [63]. The real-time assessment will benefit from further research into temporal smoothing strategies, sequence-level modelling approaches that explicitly capture transitions [64], and personalised models to account for inter-subject variability; in addition, integrating multimodal physiological signals may further enhance discrimination between closely related pain states. Another important direction is personalisation, because inter-individual variability remains a major barrier to generalisable physiological pain models; this is consistent with broader recent discussions of AI-enabled personalised pain management [65]. The inclusion of MobileNetV3 provides a representative comparison with modern lightweight architectures; however, a comprehensive benchmark of efficiency-optimised models remains beyond the scope of this study. In addition, future studies should evaluate these models outside controlled laboratory settings, since recent reviews of AI for pain assessment highlight the need for stronger validation in practical clinical environments and more realistic deployment scenarios [66]. This means that progress will likely depend not only on improving model architecture, but also on combining complementary biosignals, adapting to subject-specific physiology, and validating systems under real operating conditions.

## 5. Conclusions

This study investigated automatic pain estimation using EDA signals with both classical machine learning and DL approaches, with the objective of evaluating their suitability for real-time physiological monitoring. The results showed that reliable discrimination between three pain levels can be achieved using EDA alone, confirming that autonomic responses provide meaningful information for objective pain assessment. Among the evaluated models, the FCN achieved the best balance between classification performance and computational cost, which enabled its integration into a real-time graphical interface for continuous estimation. The GUI experiments demonstrated that although offline classification accuracy is high, real-time prediction introduces additional variability due to the gradual and dynamic nature of electrodermal responses, highlighting the importance of evaluating pain recognition systems under realistic operating conditions.

Despite these promising results, several limitations remain, including the use of a single physiological modality, controlled experimental conditions, and a global model across subjects. These factors may limit generalisation to real clinical scenarios where physiological responses are more variable. Future work should therefore investigate multimodal sensing, subject-adaptive models, and validation in real-time and real-world environments to improve robustness and practical applicability. Overall, the findings support the feasibility of using DL architectures for real-time pain estimation from physiological signals and contribute to the development of objective monitoring systems for applications where reliable self-report is not available.

## Figures and Tables

**Table 1:** Summary of the EDA pain intensity data from the AI4Pain dataset.

Category	Details
Sampling rate	100 Hz
Number of subjects	65
Number of classes	3
Number of observations for NP	715
Number of observations for LP	780
Number of observations for HP	780
Length of each observation	10 s
Observations per subject	35 (11 NP, 12 LP, 12 HP)
Total observations in the dataset	2275

**Table 2:** Selected hyperparameters for classical machine learning models.

Model	Hyperparameter	Selected Value
LDA	Solver	SVD
LR	Penalty	l2
	C value	97.2
	Solver	Newton–Cholesky
	l1 ratio	0.07
SVM	Kernel	Polynomial
	Degree	4
	Kernel coefficient	Auto
AdaBoost	N Estimators	227
	Learning Rate	0.78
GBoost	N Estimators	438
	Learning Rate	0.52
	Criterion	Squared Error

**Table 3:** Results of the evaluated machine learning models based on the median-performing fold, with performance metrics reported as mean ± standard deviation across cross-validation folds. W. denotes weighted.

Model	Best Fold	Accuracy	F1	W. Precision
LDA	2	0.75 (±0.02)	0.75 (±0.02)	0.77 (±0.02)
LR	2	0.75 (±0.02)	0.75 (±0.02)	0.77 (±0.02)
SVM	1	0.75 (±0.02)	0.75 (±0.02)	0.76 (±0.03)
ADABOOST	1	0.74 (±0.04)	0.74 (±0.04)	0.76 (±0.04)
GBOOST	4	0.74 (±0.01)	0.74 (±0.01)	0.75 (±0.01)

**Table 4:** Selected hyperparameters for DL models.

Model	Hyperparameter	Selected Value
CNN	Optimiser	Adam
	Learning rate	∼$1.62e^{-4}$
	Scheduler	ExponentialLR
	Number of CL	2
	CL0 Out channels	40
	CL0 Kernel	4
	CL0 Activation	none
	Pool0 Type	Average
	Pool0 Kernel	6
	CL1 Out channels	109
	CL1 Kernel	4
	CL1 Activation	ReLu
	Pool1 Type	Max
	Pool1 Kernel	6
	FC Layers	2
	FC0 Out Channels	46
LSTM	Optimiser	Adam
	Learning rate	∼$8.4e^{-4}$
	Scheduler	ExponentialLR
	Layers	1
	Hidden Size	15
	Dropout	not applicable
	Direction	Unidirectional
CNN-LSTM	Optimiser	AdamW
	Learning rate	∼$5.14e^{-4}$
	Scheduler	none
	Number of CL	3
	CL0 Out channels	41
	CL1 Out channels	99
	CL2 Out channels	78
	Transition	Flatten
	LSTM Layers	1
	Hidden Size	50
	Direction	Bidirectional
Transformer	Optimiser	Adam
	Learning rate	∼$1.12e^{-5}$
	Scheduler	ExponentialLR
	Encoder Layers	3
	Attention Heads	6
	FF Dimension	3
	Dropout	0.21
FCN	Optimiser	Adam
	Learning rate	∼$1.94e^{-4}$
	Scheduler	ReduceLROnPlateau
	Number of CL	2
	CL0 Out channels	71
	CL1 Out channels	120
	AvgPool Kernel	3

**Table 5:** Classification performance of the median-performing fold for each DL model. Results are reported as mean ± standard deviation across cross-validation folds. W. denotes weighted precision.

Model	Best Fold	Accuracy	F1	W. Precision
CNN	4	0.77 (±0.04)	0.77 (±0.04)	0.78 (±0.03)
LSTM	5	0.64 (±0.11)	0.64 (±0.11)	0.65 (±0.11)
CNN-LSTM	2	0.74 (±0.02)	0.74 (±0.03)	0.74 (±0.03)
Transformer	5	0.74 (±0.08)	0.74 (±0.08)	0.75 (±0.08)
FCN	4	0.79 (±0.03)	0.79 (±0.03)	0.80 (±0.03)

**Table 6:** Computational cost of the evaluated classical machine learning models. Inference time is reported as mean ± standard deviation per sample. Feature extraction required 3.77 ms per window on average across all windows from the 65 subjects. Total latency was computed as the sum of feature extraction cost and model inference time.

Model	Inference Time (ms)	Total Latency (ms)
LDA	0.01 (±7 × 10^−4^)	3.78
LR	3 × 10^−3^ (±1 × 10^−3^)	3.77
SVM	0.01 (±1 × 10^−3^)	3.78
AdaBoost	0.73 (±0.12)	4.50
GBoost	0.06 (±0.01)	3.83

**Table 7:** Computational cost of the evaluated DL models, reported as multiply–accumulate operations (MFLOPs) and inference latency per sample. For DL models, feature extraction is performed within the network during the forward pass, therefore the reported latency corresponds to the full end-to-end processing time. Latency is reported as mean ± standard deviation.

Model	MFLOPs	Total Latency (ms)
CNN	6.43	0.44 (±0.02)
LSTM	121.95	0.28 (±0.02)
CNN LSTM	3659.15	0.84 (±0.02)
TRANSFORMER	9.06	2.29 (±0.06)
FCN	52.25	0.47 (±0.02)

**Table 8:** Final architecture of the FCN model used in the GUI-based real-time estimation, showing the sequence of convolutional blocks, pooling, and flattening operations together with the corresponding input and output tensor shapes.

Layer	Sub-Layer(s)	Input Shape	Output Shape
CL 0	Convolutional	[B,1,1000]	[B,71,999]
	Batch normalisation		
	ReLU		
CL 1	Convolutional	[B,71,999]	[B,120,997]
	Batch normalisation		
	Tanh		
Avg. Pool		[B,120,997]	[B,120,332]
Flatten layer		[B,120,332]	[*B*, 39840]

**Table 9:** Comparison with previous studies using the AI4Pain dataset for three-class pain classification on the hidden test set.

Reference	Sensor Modality	Model Type	Acc. (%)
[42]	EDA, BVP, RESP, SpO_2_	TabPFN	60.06
[43]	BVP	XGBoost	62.93
[44]	EDA, BVP, RESP, SpO_2_	Tiny-BioMoE	54.89
[17]	EDA, BVP, RESP, SpO_2_	SVM (RBF kernel)	52.30
[45]	RESP	Cross-Attn. Transf.	42.24
[46]	EDA, BVP, RESP, SpO_2_	MLP	56.03
[47]	EDA, BVP, RESP, SpO_2_	TCN	59.48
[48]	EDA	Vision Transf.	55.17
[49]	EDA, BVP, RESP	XGBoost	70.11
[50]	EDA, BVP, RESP, SpO_2_	Ensemble Learn.	49.13
[51]	EDA, BVP, RESP, SpO_2_	Random Forest	62.07
[52]	EDA	CNN	55.70
[53]	EDA, BVP	CrossMod-Transf.	75.83
[54]	EDA	GIAFormer ^1^	51.62
[55]	EDA, BVP	Ensemble Learn. ^1^	74.50
[56]	EDA	MDNet 1D-CNN ^1^	71.91
This work	EDA	FCN ^2^	79.23

^1^ Leave-one-subject-out cross-validation (LOSO). ^2^ 5-fold cross-validation (5F-CV).

**Table 10:** Comparison of studies performing real-time physiological pain assessment.

Reference	Sensor Modality	Subjects	Model	Task	Validation	Latency	Performance
[57]	EDA	36	Stacked LSTM	No Pain, Pain	Hold-out	N/A	75.3% F1
[56]	EDA	65	MDNet (CNN)	No Pain, Low Pain, High Pain	10-fold CV	119.2 ms	69.84% Acc.
[58]	EDA, ECG	87	Attn. Network	No Pain, Pain	LOSO	N/A	87.06% Acc.
[59]	EDA, HR, Pupil	42	Transformer	No Pain, Pain	Hold-out	≈5.7 s	0.90 AUC
**This work**	EDA	65	FCN	No Pain, Low Pain, High Pain	5-fold CV	**0.47 ms**	**73.14% Acc.**

## Data Availability

The AI4Pain dataset used in this study is available via the official project website (https://sites.google.com/view/ai4pain2025, accessed on 7 May 2026) and can be accessed upon request in accordance with the dataset’s End User License Agreement (EULA).
